# Rose-Colored Answers: Neuropsychological Deficits and Patient-Reported Outcomes after Stroke

**DOI:** 10.3233/BEN-2009-0250

**Published:** 2010-06-10

**Authors:** Anna M. Barrett

**Affiliations:** DirectorStroke Rehabilitation ResearchKessler Foundation Research CenterAssociate Professor of Physical Medicine and Rehabilitation/ Neurology and NeurosciencesUniversity of Medicine and DentistryNJ Medical SchoolNJUSA

**Keywords:** Quality of life, anosognosia, measurement validity, self-report, stroke outcomes

## Abstract

Patient-reported, subjective outcomes are promoted as a standard for ethical, valid studies in many neurological disorders. Such outcomes are considered potentially more sensitive and specific to important therapeutic effects, and may be more linked to disability and disease-related life losses than conventional assessments of impairment (e.g. ability to walk, performance on language tests, serological or radiological indices). Self-report is invaluable to identify social and emotional consequences of brain injury: depression, changes in intimate and family relationships, social role and community participation losses. However, common stroke-related neuropsychological deficits are likely to confound subjective stroke outcome measures. The scientific community focused on stroke-related health outcomes may arrive at significantly underestimated patient reports of stroke-related disability, caused by a failure to adjust for the effect on self-report of spatial neglect, deficits of magnitude estimation, pathologic alteration of self-awareness, and alteration in distributed cortical systems supporting emotional semantics and abstraction.

## 1. Introduction

Research standards in the 21st century call for building patient-reported, subjective outcome measures, as a standard for ethical, valid studies in many neurological disorders. Patient experience is considered a more sensitive and specific measure of important therapeutic effects, and self-report may reveal disability and disease-related life losses much more accurately than conventional assessments of impairment (e.g. ability to walk, performance on language tests, serological or radiological indices). Self-report is the gold standard for measurement of many social and emotional consequences of brain injury, including depression, changes in intimate and family relationships, and adverse changes in social role and community participation. However, common specific stroke-related neuropsychological deficits are likely to confound subjective stroke outcome measures. The scientific community focused on stroke-related health outcomes may arrive at significantly underestimated patient reports of stroke-related disability. This problem may be caused by a failure to adjust for the effect on self-report of 1) spatial neglect, 2) magnitude estimation deficits, 3) pathologic alteration of self-awareness, and 4) alteration in distributed cortical systems supporting emotional semantics and abstraction.

## 2. Getting information from the “promised” source

In a recent address to the International Neuropsychological Society, researcher Jason Brandt pointed out

ISSN 0953-4180/09,10/$17.50 © 2009/2010 – IOS Press and the authors. All rights reserved that articles on patient-reported quality of life grew from fewer than ten published annually to publications in the thousands today [10]. An Ovid Medline search for 2008 articles on quality of life outcomes identified $>$ 8,000, most using self-reported outcomes (OvidSP, WoltersKluwer Health; searched May 24, 2009). Investment in patient-reported outcomes has been extensive. The National Institutes of Health (NIH), under the NIH Roadmap theme of reengineering the clinical research enterprise (http://nihroadmap.nih.gov/), identified better assessment of PROs as a pressing need, and this was realized on the "big science" scale when a cooperative US network was funded in 2004. This initiative, the Patient-Reported Outcomes Measurement Information System (PROMIS; see www.nihpromis.org), receives multi-million dollar annual NIH investment for development of standard, flexible means of assessing subjective patient reports.

Patient-reported data is central to the meaning and purpose of medical research. Symptoms of a disease or disorder are not the experience of illness, and it is the experience of illness that drives people affected by a condition to seek healthcare, and receive treatment. Investigating the experience of illness more directly is tremendously important, as are better methods of exposing and measuring the limitations disease imposes on life.

Health researchers have thus turned with new enthusiasm and commitment to self-report data to measure stroke outcomes. Over the last 40 years (see Sarno and Gainotti [33], for a review), a number of leading researchers demonstrated that adverse social and psychological consequences occur after stroke, some of which are common to any loss, and some of which may be stroke-based (e.g. altered self-image with cognitive or physical changes). These include depression [22], alteration in the caregiver-survivor relationship [30] and pathologic alteration of the survivor's social and family role. Treatment research evaluating impairment as a primary dependent measure will not be sensitive to these changes, nor to the interaction of these changes with appropriate rehabilitation.

An obvious example of how the field may benefit from patient-reported outcomes in stroke care is in measuring the effect of compensatory strategies on health outcomes. Such interventions (e.g. a power wheelchair), which do not alter a stroke survivor's neurological condition, may still improve the survivor's ability to resume her role in the community. In contrast, measuring impairment-based variables (e.g. leg strength) may result in a false conclusion that no change in health status occurred. Laudably, PROMIS and other "quality of life" research studies include previously-underemphasized domains for assessment (e.g. fatigue and emotional distress) and develop theoretically eloquent social assessment domains (distinct consideration of social health, function and support). Self-report research initiatives thus have the potential of providing better instruments with which to study social and life function in the setting of medical illness.

## 3. Neuropsychological disorders confound subjective data

It would be wonderful to collect self-reported outcome measures to assess outcomes in stroke survivors, if not for a considerable number of stroke-related neuropsychological factors. The confounding influence of these very common conditions may make it impossible for survivors to report their experience accurately, and may result in data systematically overestimating wellness. Survivor difficulty in reporting degree of distress, disability, or even impairment accurately due to these neuropsychological problems may be completely independent of intelligence, intentions, or the sophistication and subtlety of development, in normed matched healthy controls or proxies, of tests used to measure their responses. This critical lack of validity in patient-reported outcome measurements after cortical injury is rarely discussed or addressed.

In this article, I will not explore concerns about how widely adopted invalid research methods would affect our science or our field (see eloquent commentary on this issue by Brandt [10]). I also will not discuss methods of adapted data collection in stroke survivors with communication disorders, as aphasia is frequently acknowledged as posing a challenge to patient self-report (e.g. Dorman et al. [15]). Rather, I will present four neuropsychological influences we can expect may systematically alter subjective self-report of the experience of illness. My overview of these influences is brief; I encourage readers interested in these phenomena to explore the extensive literature available in each area.

Based on reports assessing the frequency of similar neuropsychological conditions [6,17], it is possible that one of two stroke survivors (50%) overestimates self-function due to at least one of these problems. By increasing attention to these confounds, we may be able to resist the temptation to assess quality of life and other self-reported outcomes in stroke, in a manner better suited to the cognitively healthy, and thus avoid an influx of data of uncertain value and relevance. I end this article by making some summary recommendations on how targeted research might improve valid inquiry in this important area of health outcomes.

## 4. Spatial neglect

A first major cause of overestimated self-function in a stroke survivor is post-stroke spatial neglect. This disorder, a pathologic failure to report, respond or orient to stimuli in contralesional space [27], causing functional disability [7], may occur in as many as 80–85% of right hemisphere stroke survivors depending on how it is assessed [1,28,37], and at least 20–30% of right hemisphere survivors have moderate-severe, clinically significant symptoms [1,12]. Acute severe spatial neglect symptoms are dramatic (head and eye deviation to the ipsilesional side, failure to move into contralesional space or acknowledge stimuli in that region), and it might thus seem unlikely that unsuspected effects of spatial neglect may occur in self-reporting. However, it has been demonstrated that right hemisphere stroke and spatial neglect is under-recognized [16,20]. This might be more likely when patients manifest some, but not all typical spatial neglect symptoms, or when spatial neglect symptoms are milder. The typical, 1-to-5, visual analog or Likert scale cannot be marked accurately when a subject has spatial neglect: he or she will overuse the right end of the scale. Although it may less affect vertical-scale responses, spatial neglect also induces upward bias (lower-page neglect), and can be expected to induce overuse of the top of a self-report scale (e.g. Shelton et al. [34]).

## 5. Anosognosia and anosodiaphoria.

If visual-spatial bias were the only consideration in stroke survivors, balanced response instruments (with the high end of a Likert scale alternating on the left and right, or top and bottom) might assist with obtaining valid data. However, stroke survivors with either left or right hemisphere stroke may also experience pathologic unawareness of deficit (anosognosia; Adair et al. [3]). Unawareness of hemiparesis, occurring in the days and weeks following stroke and undetectable after a few months, is a classical example: the survivor may report that the paralyzed arm is “lazy”, or may express dislike or distorted perceptions relating to the paralyzed arm, but deny arm weakness. These symptoms are dramatic in severely impaired patients, particularly after right hemisphere stroke. However, many clinical researchers are not aware that anosognosia for impairments of memory, visual-spatial function, language ability, and visual acuity may also occur in stroke survivors without spatial neglect or reality distortion [36], and the problem may occur in certain functional domains, leaving others unaffected [5]. Indifference to the impact of a deficit (anosodiaphoria or “lack of suffering from disease”) may also occur after stroke [3] and is felt to be closely associated with anosognosia. Its prevalence is not known, as it is rarely specifically assessed. Stroke survivors may thus be expected to overestimate function in stroke-affected domains, which may result in under-reporting of the impact of stroke on daily function or quality of life. This may occur even where ability to estimate and self-estimate in other areas is relatively intact. Anosognosia is associated with poor stroke rehabilitation and recovery [23,25], and even mild unawareness may adversely affect stroke outcomes, since survivors capable of higher-level function attempt to resume more complex, risky life activities (e.g. driving).

Anosognosia and anosodiaphoria, like spatial neglect, are perceived to be rare, but may affect at least a third to one-half of stroke survivors (a number comparable to stroke survivors with cognitive challenge; Barrett and Rothi [6]). Clinicians have casually observed that family members frequently report that survivors over-estimate when asked about social participation and other life experiences, and thus this problem may be a serious methodological challenge to self-report assessment. In a recent large study of the impact of visual impairment, for example, people with comorbid stroke may have indicated much less impact of vision loss on quality of life than those with other disease states [11]. Although anosognosia may improve spontaneously during stroke recovery [28], clinicians frequently report that unawareness interferes with functional competence (for example, return to work; Sherer et al. [35]) in the chronic brain injury recovery phase, even years later.

Identifying stroke survivors with impaired awareness of stroke-related deficits will not only result in improved quality of self-report data from groups of stroke survivors – it could benefit individuals after stroke. Behavioral (explicit reminding of discrepancies between self-assessed and objectively measured performance; implicit techniques in which subjects are guided to experience the contrast between preserved abilities and deficits; see Eslinger et al. [19] for a review) and physiologic treatments (e.g. vestibular caloric stimulation of right hemisphere awareness systems; Cappa et al. [13]) may improve anosognosia after brain injury. Initial studies suggest this treatment might in turn improve functional outcomes [24]. Routine clinical assessment of anosognosia after stroke is needed to stimulate further investigation of these treatments. Until stroke survivors with anosognosia are routinely identified, we also have no means to make treatment for this symptom available.

## 6. Abnormal magnitude estimation

The ability to report the severity of a problem requires the cognitive skill of magnitude estimation – the ability to judge how one event, experience or stimulus compares with another. Mennemeier and colleagues demonstrated in a series of elegant studies that magnitude estimation may be abnormal after stroke [31, 32,38,41]. In their reports, independent of intellectual, mathematical, analytical or communication ability, and independent of an accurate internal experience of loss, a stroke survivor may have difficulty with indicating "how big," "how bright," "how loud," or, in the case of quality of life or stroke impact, "how serious." Right hemisphere stroke survivors systematically report events of moderate or increased magnitude (for example, a bright light), as being of lesser magnitude (less bright) than do controls. This is a different cognitive phenomenon than spatial neglect, because when events are of small magnitude (a dim light), errors switch direction, and the stroke survivor may over-report event magnitude (brightness). Mennemeier and colleagues reported that the degree of visual-spatial estimation error in right hemisphere stroke survivors might be predictable by a simple formula based on Steven's power law. However, their research also suggests that the problem is not limited to overestimation – in survivors of left-hemisphere stroke, pilot findings reveal errors under-estimating self-function, with survivors reporting strength in the affected limb to be more disabled than objective measures indicate. Because self under- or over-estimation of function may affect how survivors reach goals during rehabilitation, the National Institutes of Health awarded this group a current research grant to examine whether self-function estimation errors correlate to rehabilitation outcomes.

## 7. Deficits affecting semantics and abstraction

Lastly, stroke may impair the critical cortical regions supporting internal emotional experience, representations or concepts of the subjective problem/function being assessed (emotional semantics; Blonder et al. [9]). For example, just as a never-married person or a child might have difficulty understanding the experience of marital dysfunction, a person with a brain injury affecting the formation or maintenance of meaning systems (semantics, critically supported by distributed neural systems in posterior association cortex), may not be able to access all aspects of his or her internal experience of depression, pain, fatigue, emotional distress, social dysfunction, or a number of other important experiences. The resulting superficial conscious awareness may lead the survivor to under-report negative emotional states he or she is experiencing, which are essential to understanding disability and challenge in stroke recovery.

This disorder is particularly characteristic of injury to the right cerebral hemisphere, or frontal-limbic connections. Higher brain centers have been known to be important to accurate representation, experience and reporting of depression and pain for more than 50 years. Although not affecting basic pain receptors or mood, prefrontal lobotomy for intractable pain and depression [21,40] appeared to relieve patient suffering. Distinct from anosodiaphoria and anosognosia, disruption of frontal-limbic pathways reduces the survivor's access to personal emotional experience and personal emotional meaning (see Eslinger and Damasio [18] for a classic case report, in which the disorder followed brain tumor removal). As Freeman wrote, the "relationship of the self with the self" critical for successful introspection, is physically maintained within the brain, and can be removed by limbic cortical disconnection.

Assigning a rating also requires the capacity for abstract thinking. As we answer a questionnaire about our perception of the consequences of a medical event, we may not be aware that we are making several complex judgments: that each item should be answered separately; that there is no "right" or "wrong" answer; that a specific internal state exists called "depression," "pain," and "satisfaction." We know that some people may be unfamiliar with how to make these judgments because of limitations of their premorbid education, cultural or intellectual background. However, many clinical researchers may not know that the ability to make these mental judgments can be adversely affected after stroke, in people who do not meet criteria for dementia. Because the process of their thinking, rather than the content of their thinking, is affected, cognitive limitations may go undetected by medical professionals.

## 8. Recommendations for research progress

There are several steps needed to improve the validity of self-reported research data in stroke survivors with the neuropsychological deficits noted above. The most important step is to act to improve the odds that relevant neuropsychological deficits are routinely detected during clinical care (e.g. spatial neglect, anosognosia). This is only effectively accomplished by appropriate education and training to clinicians. General health screening tools, or tools to assess global neurological function, are likely to be poorly sensitive to cognitive deficits (e.g. the NIH Stroke Scale is poorly sensitive to spatial neglect; Hillis et al. [29]; Cumming et al. [14]). For spatial neglect, specific training to improve the ability of stroke physicians, nurses, and therapy personnel to detect spatial errors by observing patient behavior during ward activities may improve detection of this syndrome. Using specific cognitive tests to screen for neuropsychological deficits is also a good option, but this is time-intensive as examiners must be well trained. A screening instrument such as the Catherine Bergego Scale [2], for example, is used to screen all stroke survivors, in a clinical-research partnership program between our center and a clinical rehabilitation institute. Neglect screening helps to direct rehabilitation, as well as providing access to treatment research [8]. However, this routine screening with a standardized assessment instrument requires that clinicians have an excellent fundamental understanding of typical manifestations of the disorder, and also requires specific time investment in clinician reliability training.

As we work on the larger problem of improving clinician assessment of spatial neglect, we could reduce confounding by spatial neglect of Likert scale responses, by routinely right-left and up-down balancing patient responses. Response keys should be weighted so as to flag consistent rightward/upward responses, thus allowing this potential evidence of a spatial cognitive disorder to be reported back to clinicians for appropriate further assessment and care.

With regard to anosognosia, it is possible that adding "catch trials" – questions to which aware stroke survivors would respond positively, reporting difficulty – might increase the ability to co-evaluate response validity. There is a model for this in subjective questionnaires such as the Minnesota Multiphasic Personality Inventory (MMPI; Hathaway and McKinley [26]). The MMPI includes items (L or Lie Scale) intended solely to evaluate whether the subject is under-reporting problems (e.g. "I get angry sometimes" – false). Development of similar items for validity assessment in stroke quality of life reporting might be helpful in interpreting disparities between the reported impact of a post-stroke neurological problem, and its measured severity.

As summarized in the above section, researchers evaluating magnitude estimation impairment after stroke suggested that magnitude estimation errors might actually be mathematically predictable [31,32]. If so, it is possible that by pre-evaluating a stroke survivor's ability to estimate physical stimulus parameters (brightness, loudness, length), clinicians might be able to derive and apply a "correction factor" to obtain accurate magnitude estimation of patient-reported subjective quantities (e.g. quality of life). Specific research studies would be needed to test this concept and approach.

Detecting a confound induced by inability to experience emotional meaning, or apprehend and use abstract information, again demands that stroke caregiving develop a broader on thinking, including metacognition and thought process. Educating medical and therapy professionals about how stroke can adversely affect emotional processing – in particular, how these problems are distinct from typical psychological reactions to illness such as denial, rationalization or minimization – is tremendously important. Questionnaires used experimentally to detect deficient understanding of emotionality after stroke (e.g. Blonder et al. [9]) might be useful correlate measures in quality of life self-reporting. However, to develop such a procedure we would need research specifically supporting a relationship between the ability to self-report emotions to standardized stimuli, and self-report of quality of life, and these studies have not yet been performed.

It will be similarly challenging to account for stroke-related problems with understanding and manipulating abstract concepts as a cause of abnormal self-reported quality of life, because abstract thinking is often confused with premorbid intelligence by healthcare professionals. Unfortunately, this means that people with excellent intellectual ability, who nevertheless have problems with abstract thinking, are not usually identified. Educating health professionals about how stroke affects thinking could be incorporated into stroke quality care certification for practitioners, units, or hospitals, and this might improve the likelihood that healthcare providers would test for "hidden disabilities" affecting mental function. Once abstract thinking problems are routinely identified, it becomes feasible to focus research on using abstraction assessment [4] to validate quality of life reporting, identify stroke survivors unlikely to give accurate self-reports, or even explore a "correction factor" which might be applied to self-report data in this context.

## 9. Conclusions

In this article, I reviewed and briefly described four neuropsychological disorders: spatial neglect, anosognosia and anosodiaphoria, magnitude estimation deficit, and problems with emotional semantics and abstraction. Each of these disorders may be present in a high-functioning, verbal stroke survivor expected to be intellectually competent, and would induce systematic distortion of self-report data. Specifically, each disorder would cause affected survivors to overestimate well-being and level of function, and underestimate stroke impact.

As mentioned in the Introduction, other stroke-related cognitive abnormalities impair self-reporting. I have deliberately not discussed aphasia, which as a disorder of speech and language, inherently impairs the ability to use language and symbols to communicate internal experience. Although this is an obstacle to collecting patient-reported outcomes, the traditional solution is to exclude stroke survivors with aphasia from this kind of research. This bias is troubling, and needs to be re-examined. However, exclusion of a stroke survivor subgroup is different from inclusion of potentially invalid data, and thus I did not attempt to discuss the issue of developing and using adapted communication methods for survivors with aphasia, which is vitally important.

Translational neuroscience can be interpreted narrowly, as the means by which bench research leads to new pharmacological approaches to neurological disease. Perhaps the most daunting challenge of translational cognitive neuroscience, however, is translating and applying developed concepts across fields and in new ways. Such an endeavor requires the translator to surmount obstacles in scientific social culture and assumptions. Attempting to acknowledge and integrate neuropsychological threats to validity of self-report data in the area of quality of life, treatment outcomes, and health in stroke requires enormous effort, and potentially requires restructuring small- and large-scale assessment research. Is the effort worth the cost? It is, only if we appreciate and wish to preserve the value of the stroke survivor's experience. As this experience is the root of the best discoveries in cognitive neurology–indeed, in medicine–I think we know the answer.

## Acknowledgements

This work was funded by the National Institute of Neurological Disorders and Stroke (5K02NS047099-06 and 5R01NS055808-02; Barrett, PI) and the Kessler Foundation.
